# Simulating the Intraoral Aging of Dental Bonding Agents: A Narrative Review

**DOI:** 10.3390/dj10010013

**Published:** 2022-01-15

**Authors:** Tomas Vilde, Cameron A. Stewart, Yoav Finer

**Affiliations:** 1 Faculty of Dentistry, University of Toronto, Toronto, ON M5S 1A1, Canada; tomas.vilde@mail.utoronto.ca (T.V.); cameron.stewart@mail.utoronto.ca (C.A.S.); 2 Institute of Biomedical Engineering, University of Toronto, Toronto, ON M5S 1A1, Canada

**Keywords:** dental bonding agents, dental material testing, aging, biodegradation

## Abstract

Despite their popularity, resin composite restorations fail earlier and at higher rates than comparable amalgam restorations. One of the reasons for these rates of failure are the properties of current dental bonding agents. Modern bonding agents are vulnerable to gradual chemical and mechanical degradation from a number of avenues such as daily use in chewing, catalytic hydrolysis facilitated by salivary or bacterial enzymes, and thermal fluctuations. These stressors have been found to work synergistically, all contributing to the deterioration and eventual failure of the hybrid layer. Due to the expense and difficulty in conducting in vivo experiments, in vitro protocols meant to accurately simulate the oral environment’s stressors are important in the development of bonding agents and materials that are more resistant to these processes of degradation. This narrative review serves to summarize the currently employed methods of aging dental materials and critically appraise them in the context of our knowledge of the oral environment’s parameters.

## 1. Introduction

Dental caries are one of the most common maladies in the human population today, affecting 35% of the global population across all ages in 2010 [1], with peaks of cavitated dental caries incidence at the ages of 6, 26, and 70 years old [2]. This presents a significant burden on the healthcare system, and barring prevention or early detection, treatment is required to remove the decayed tissue and restore the lost tooth structure with a restorative material. The most common restorative materials are resin composite restorations, due to their improved aesthetic appearance, and versatility in comparison to older extant materials [3]. However, these improvements came at the cost of worse performance and outcomes in comparison to amalgam restorations; resin composites have an annual failure rate as high as 12.9% in primary teeth [4], and in some cases, the service life of a resin composite restoration has been found to be half as long as an amalgam one, requiring more frequent repair [5,6,7]. This has been an ongoing problem, as found in a 2015 systematic review by Moraschini et al., which found that posterior composite restorations still have less longevity, and a greater likelihood of failing due to secondary caries in comparison to amalgams [8]. The quality of resin composite restorations is highly technique sensitive [7]. For example, differences in the curing method significantly affect marginal adaptation, bacterial biofilm microleakage, and caries formation and/or detachment of the restoration [9]. These possible vulnerabilities have driven research and innovation to develop resin composite materials with improved properties, but these need to be assessed in a laboratory setting before their clinical assessment and applications are considered. 

Resin composites are bonded to the tooth structure by means of a dental bonding agent, as the resin composite is too viscous, and its monomers too bulky, to penetrate the dentin. The role of the bonding agent is therefore to chemically and micromechanically bind both the underlying tooth tissue and the overlying restoration, effectively knitting the two together [10]. The quality of this bond is critical for the performance and longevity of the restoration [11], and the deterioration of this bond is more likely to result in critical failure (restoration detachment, fracture) than deterioration of the restoration bulk.

Though clinical trials remain the ideal method to study the performance of new dental materials and procedures by testing in the exact environment they are meant to function in, such trials can be difficult to conduct due to many factors, including dental materials’ sensitivity to operator experience, patient compliance, and the expense and time required to conduct the trial for numerous formulations during development [12]. In vitro tests are therefore a valuable way to estimate the viability of a material in an easy and rapid manner.

This narrative review aims to review the state of current laboratory protocols that are in use to test dental adhesives, critically evaluate them in the context of our knowledge of the oral environment and suggest means for improvement.

## 2. Chemistry of Resin Composites

A resin composite makes up the bulk of the restoration and is the overlying material meant to replace the lost tooth structure. Resin composites are made of two components: first, a polymer network, generally made of methacrylate monomers, and a particulate filler that makes up most of the material by weight [13]. Fillers are incorporated in order to improve the mechanical properties of the resulting composite, and increased filler content has been found to decrease the resin’s susceptibility to chemical degradation [14].

### 2.1. Chemistry of Dental Resin Monomers

**Figure 1:**
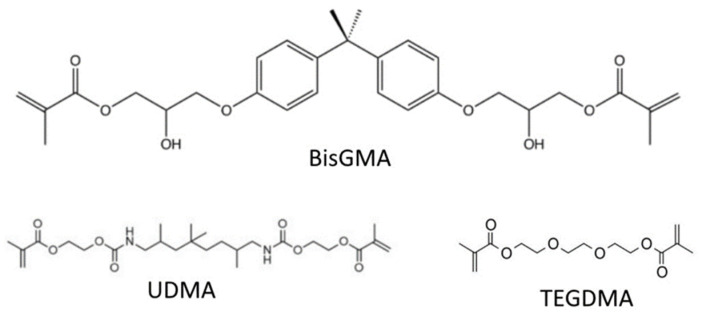
Common crosslinking monomers in dental resin composites.

Bisphenol A-glycidyl methacrylate (BisGMA) is one of the most common resin composite monomers due to its ability to crosslink, its hydrophobicity, and the rigidity it imparts on the polymerized structure (Figure 1), though many others are also commonly used [11,12]. The reason for the incorporation of other dimethacrylate monomers is due to the high viscosity of unpolymerized BisGMA, due to its pi-pi bond stacking, and high level of hydrogen bonding. This high viscosity can both inhibit the incorporation of fillers that help to both strengthen the material and impart color on it and can also stop the mobility of monomers within the mixture, decreasing the degree of conversion of the monomers into the polymer network. Diluent monomers with lower molecular weights, such as triethylene glycol dimethacrylate (TEGDMA), and urethane dimethacrylate (UDMA), are, therefore, necessary (Figure 1). Incorporating too great a proportion of such relatively hydrophilic monomers, however, increases the absorption of water, and due to the smaller molecule size, can cause excessive shrinkage of the restoration during curing [15,16,17].

These methacrylate resins polymerize by means of a vinyl reaction, forming a polymethacrylate backbone with ester linkages between backbone polymers that create a stiff matrix [18]. This polymerization is usually triggered by a photochemical initiation system activated by blue light (468 nm) [16], or a chemical initiation system, either of which is also incorporated into the material [19].

### 2.2. Bonding Agents 

Resin composites are bonded to the tooth structure by means of a dental bonding agent, as the resin composite is too viscous, and its monomers too bulky, to penetrate enamel and dentin. The role of the bonding agent is therefore to chemically and/or micromechanically bind both the underlying tooth tissue and the overlying restoration, effectively knitting the two together [10]. These bonding agents polymerize by the same mechanism that resin composite monomers do, and often use the same monomers as the overlying composite such as BisGMA, albeit at different ratios [16]. This is required as the bonding agent and resin composite must be chemically compatible with one another to polymerize together.

Bonding to enamel is achieved by first etching the surface of the enamel in order to condition it by first clearing any organic or smear layer material, creating microscopic pores in the enamel that liquid resin can penetrate. Curing, therefore, results in a network of resin tags that penetrate the enamel and hold the resin composite in place. Enamel bonds are the more reliable method of bonding, because of enamel’s low proportion of organic material [20,21]. Such minimally invasive bonds can last upwards of 20 years, and effectively seal the resin–dentin bond from the oral environment. In deeper cavities, where an enamel bond cannot be achieved, bonding wet dentin to methacrylate monomers poses a greater challenge. 

**Figure 2:**
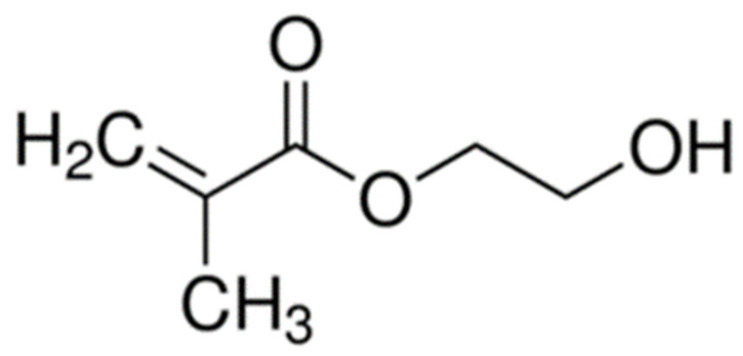
Schematic of 2-hydroxyethyl methacrylate.

In order to effectively anchor the restoration to dentin, the bonding agent must penetrate the dentin’s collagen network. Adequate penetration of the bonding agent into the underlying tooth material is accomplished first by the demineralization of the exposed dentin. Demineralization can be accomplished in two different ways. The first is by the incorporation of a phosphoric acid etching step, which frees dentin collagen fibrils from the hydroxyapatite mineral they are encased in as in total-etch or etch-and-rinse systems, or by the application of a primer that is itself acidic, as in self-etch systems, making the monomers themselves capable of demineralizing the dentin [16]. For example, the earliest self-etch system contained the acidic monomer 2-(methacryloyloxyethyl)phenyl hydrogenphosphate (Phenyl-P), wherein the hydrogenphosphate group imparts an acidic pH on the primer. Such systems have a higher pH than that of phosphoric acid etchants [22]. In the case of total-etch adhesives, after demineralization, a primer composed of bifunctional mono-methacrylate monomers, such as 2-hydroxyethyl methacrylate (HEMA) (Figure 2) [16], can be applied to the exposed collagen. Compounds such as HEMA are smaller and far more hydrophilic than BisGMA [23], and therefore, capable of diffusing into the gaps between the wet collagen fibrils and are attracted to the collagen by intermolecular forces. After priming, the bonding resin itself can be applied. The bonding agent is chemically similar to the overlying composite restoration, and they often employ similar methacrylate-based monomers, such as the bulky BisGMA that provides the mechanical strength within the resin by linking the long chains of polymer that form during curing together, both within the bonding agent and between the bonding agent and the composite [24]. Primer and bond resin can also be combined in certain systems and applied together. 

### 2.3. The Hybrid Layer

**Figure 3:**
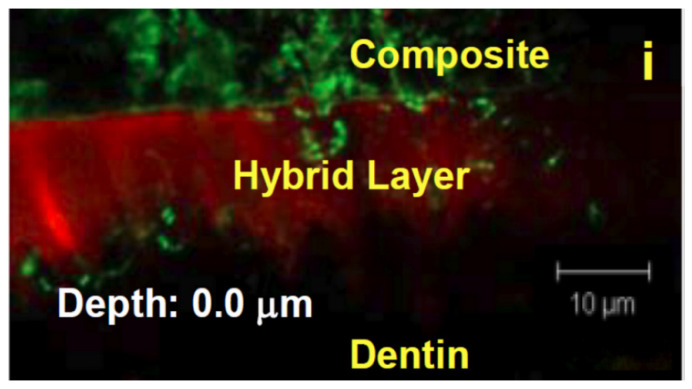
An image of the hybrid layer taken using confocal laser scanning microscopy (CLSM).

In both self-etch and total-etch systems, the application of the bonding agent results in the formation of a region known as the hybrid layer, where resin monomers interdigitate with the dentin’s collagen network (Figure 3). The hybrid layer, or resin-tooth interface, is generally considered to be the weak link of the restoration, as it is the key to the mechanical viability of the restoration as a whole, anchoring the restoration to the remaining dentin or enamel, and functioning as a stress-absorbing layer to allow the restoration to withstand mechanical stress [11,18,25]. This hybrid layer is vulnerable to degradation from a variety of sources. Often, a marginal gap forms between the composite and dentin, thus allowing saliva, bacteria, and neutrophils to penetrate the hybrid layer in a highly complex, synergistic process [26,27].

## 3. Sources of Mechanical and Chemical Stress and the Degradation of Intra-Oral Resin Composites 

### 3.1. Mechanical Degradation

Fracture is one of the most common reasons for restoration replacement, with up to 18.8% of all failures ascribed to it [28]. A fracture as a cause of failure is or matched closely with, a secondary caries, in terms of the prevalence in the cause of restoration failure [15,29]. This underlines one of the most basic requirements of a dental resin; the necessity of a hybrid layer that has the mechanical integrity to withstand years of daily chewing, grinding, etc., as the hybrid layer is considered to be the basis of the restoration’s mechanical strength [30,31]. Such cyclic loading takes place with approximately 1–2700 cycles a day, and at least 300,000 cycles over the course of a year on average [32,33,34]. This daily use results in a cyclic loading stress on the hybrid layer that can fatigue the material over the long term, slowly compromising the material through the propagation of subcritical cracks [35], and increasing the penetration of water and saliva into the interface. Such cyclic fatigue in water, for example, has been found to decrease the fracture toughness of bulk resin composites [36], but studies of the fatigue of the resin–dentin interface are far scarcer. According to one of the review articles consulted, more evaluations concerning microtensile testing of the resin–dentin interface have been published in one year than have been reported on the fatigue properties of the resin–dentin bonded interface in total [32]. Of those that exist, however, the interface has been shown to be affected negatively by a variety of factors, such as the enzymatic degradation by dentin proteases [32], and biofilm attack [37]. Thus, mechanical properties are subject to slow degradation, not only by cyclic loading fatigue, but other factors that will negatively impact this cyclic loading. This cyclic loading fatigue has recently begun to receive focus in the development of novel dental materials. Assuming an adequate application of the bonding agent, bonding agents are well suited to withstanding the mechanical stresses of the oral environment in the short term [18]; it is long term processes of fatigue and degradation that cause failure.

### 3.2. Chemical Degradation

**Figure 4:**
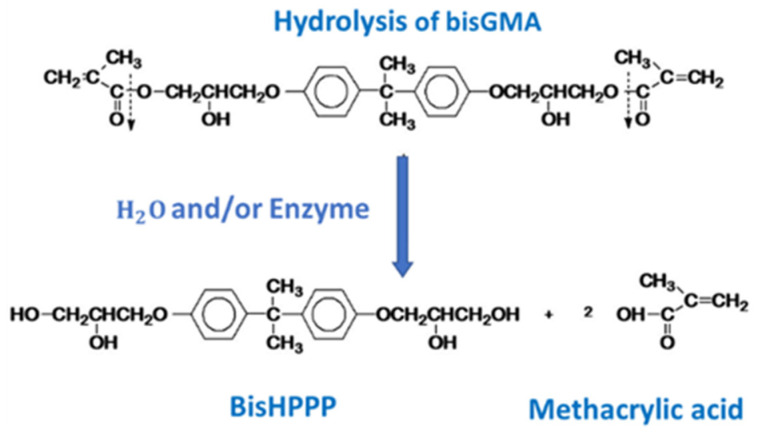
The hydrolysis of BisGMA. The carboxylic acid groups (which connect the bulk of the molecule to the ester linkages that integrate it into the resin network) are hydrolyzed, releasing the bulk of the molecule, and removing a crosslink between polymer chains.

Though mechanical degradation is the most physically obvious source of the degradation of the mechanical properties of a bonding agent, it is not the only challenge the restoration faces. Resin bonding agents are inherently not inert substances, and this makes polymerized dental bonding agents vulnerable to chemical degradation by enzymes or hydrolytic conditions within the oral cavity. Though there are a multitude of possible sources of this degradative stress [33], one significant vulnerability of these polymers is that the ester bonds of the methacrylates can be hydrolyzed in the presence of water (Figure 4) [10]. 

This hydrolysis can occur spontaneously, and is thus an unavoidable problem in the mouth, as water is both ubiquitous and a necessary component of adequate resin bonding to dentin. Acid-etched dentin should not be completely dry before applying the bonding agent, as the loss of water from the exposed collagen network could cause its collapse; this negatively impacts the penetration of adhesive into the spaces between collagen fibrils, and therefore the formation of an adequate hybrid layer [38]. This is especially true in the case of total-etch adhesives, which are more dependent on the wet bonding technique [39]. The incorporation of primers containing monomers such as HEMA in conjunction with polar solvents such as water or ethanol, is therefore, required [40]. These primers wet the dentin due to their hydrophilicity, penetrating the matrix, and displacing the water within it. However, this process of water removal is often incomplete [39,40], and the hydrophilicity of monomers such as HEMA increases the uptake of water into the resin after polymerization, affecting the mechanical properties of the resin in the hybrid layer over time. In addition, insufficient penetration of the resin monomers into the demineralized collagen network means an incomplete hybrid layer, which may also increase the penetration of water into the hybrid layer, and the hydrolysis of collagen and resin. Similarly, the relative hydrophilicity of the self-etch system attracts water into the adhesive and results in material dissolution, plasticization, and degradation [34,35]. This also does not account for the proteins and other components of saliva. Salivary, bacterial, and neutrophil esterases, for example, have been found to accelerate the degradation of BisGMA, measured by the release of 2,2-Bis [4(2,3-hydroxypropoxy)phenyl]propane (BisHPPP) from adhesive resins, at a rate significantly greater than the biodegradation previously reported in the literature [18,41]. Incubation in saliva or artificial enzyme solutions has also been found to decrease the interfacial fracture toughness of the resin–dentin interface [42], in addition to forming a network of pores and gaps, which may facilitate further water sorption and degradation and the increased penetration of bacteria into the hybrid layer [26]. 

Acidity is another commonly considered mechanism of chemical degradation, as acidity gradually demineralizes enamel and dentin. The critical pH at which enamel generally begins to dissolve is approximately 5.5, while softer root dentin may begin to dissolve at 6.7 [33,43]. The oral environment is maintained at an approximately neutral pH, in a range from 6.2 to 7.6, protecting teeth from acidity [33]. Acidity may, therefore, not be a significant concern in normal conditions as it is actively controlled by salivary flow and buffering [33]. However, an excessive intake of highly acidic or sugary food and drink may lower the oral pH and displace the buffering saliva; dental caries are formed partially due to an acidic attack on the enamel and dentin by bacteria; this changes the pH in the microenvironment around the bacterial colony, but generally not permanently in the oral environment as a whole [44]. In these scenarios and specific regions, pH may play an outsized role, accelerating the dissolution of the margins around the restoration. That being said, the specifics of this relationship between pH and interface degradation has not yet been the subject of a study [45].

### 3.3. Bacterial Degradative Properties 

**Figure 5:**
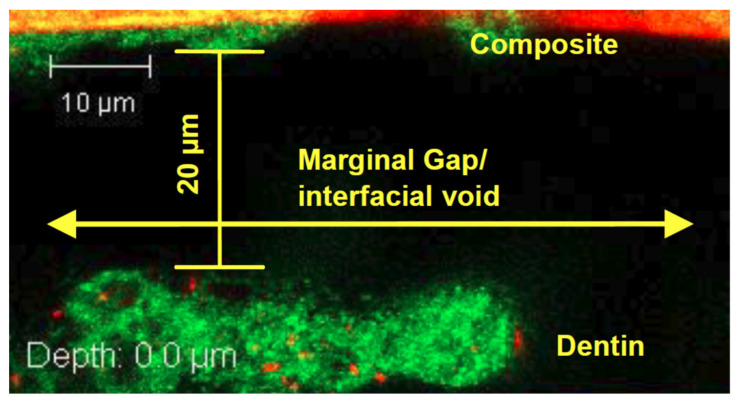
*S. mutans* biofilm present within the marginal gap between the composite restoration and dentin, as shown by the green-stained biofilm.

Bacteria are ubiquitous in the oral environment, and certain species, such as *Streptococcus mutans*, have been found to be the primary progenitors of dental caries [46]. Bacteria such as *S. mutans*, one of the primary culprits for early caries formation due to their acidogenicity and aciduricity, are also known to produce esterases capable of degrading dental methacrylate resins [47,48]. *S. mutans* is capable of penetrating the marginal gap and forming a biofilm within the void (Figure 5), and the biofilm has been found to penetrate deeper into the void the longer a bonded interface is aged in simulated salivary enzymes [26]. The presence of BisHPPP may also cause a positive feedback effect on the bacteria, as *S. mutans* incubated in BisHPPP was found to increase the expression of resin degrading esterase [47]. Both BisHPPP and tri-ethylene glycol (TEG), biodegradation by products of resin monomers, affect the expression of virulence genes and other relevant proteins in *S. mutans* [3,40,47,49,50]. In the microenvironment of the marginal gap, the diffusion of these by products occurs very slowly, potentially causing a lingering effect on the bacteria.

Besides degrading the bonding agent’s resin, cariogenic bacteria are also proteolytic [51]; therefore, once the remaining dentin and enamel at the margin have been demineralized, the bonding agent resin and the exposed dentinal collagen of the hybrid layer are both degraded, compromising the hybrid layer [27].

### 3.4. Chemical and Mechanical Deterioration of the Hybrid Layer 

These varied mechanisms of in vivo degradation act on the hybrid layer and can act synergistically to compromise the resin–dentin interface over time. For example, salivary degradation and mechanical fatigue have both been found to increase the size of the marginal gap between restoration and dentin [18], and their effects may be larger or smaller depending on the technique used in creating the restoration [9]. A larger marginal gap increases the exposure of the resin–dentin interface to the oral environment, and increases the leakage of bacteria and saliva into the hybrid layer [26,52], accelerating the degradation process and caries formation. Eventually, the hybrid layer’s shock absorbing properties may also be affected, causing higher stress concentration, and therefore, increasing the risk of restoration detachment [25]. 

## 4. Quantifying the Chemical and Mechanical Degradation of Dental Bonding Agents and Bonded Interfaces In Vitro

### 4.1. Quantification of Chemical Degradation 

A major challenge inherent in simulating the aging of dental materials in the oral environment is the quantification of the changes in the material or bonded interface. One of the most common ways to detect the chemical degradation of a dental bonding agent is the use of liquid chromatography, at times in combination with mass spectrometry to measure the release of biodegradation by products such as BisHPPP from a sample [18]. BisHPPP is a byproduct produced when the ester linkages at both ends of a BisGMA molecule are hydrolyzed (Figure 4). A standardized specimen, for example, a 4 × 4 mm cylinder of bonding agent [53], or the release from a mechanical specimen being aged chemically [54] could be quantified this way.

### 4.2. Quantification of Mechanical Degradation

From a mechanical perspective, many studies have focused on the mechanical properties and durability of the overlying resin composite that makes up the bulk of the restoration, is most exposed to the oral environment, and is the surface upon which the mechanical stresses of the oral environment impact. However, the hybrid layer is considered a key source of the mechanical strength of the restoration and a stress absorbing layer [30,31], making it, and the bonding agents that assist in forming it, vital subjects of study.

In terms of the mechanical properties of bonding agents, a general misconception when it comes to the testing of dental materials is that strength is an inherent property of the material. However, strength often depends on a number of factors such as the geometry of the sample in question or the presence of imperfections at the interface of materials, and is therefore not a constant property of a given material [55,56]. Thus, although there are many different tests that can be conducted to measure the bond strength, care must be taken to ensure that the interface being tested is free of flaws. Especially when testing large bonded areas, the validity of such “macro” (>2 mm^2^ bonded area [57]) tests must be considered, due to the prevalence of a cohesive failure rather than an interfacial failure, and also the problems of ascribing an average stress value to an interface that bears stress unevenly [58]. Loading macro specimens in shear rather than tension, for example, can result in higher stress concentrations, and the manner in which the loading of the specimen occurs may also affect the recorded bond strengths; therefore, it has been mentioned that it is important to describe specimen design and configuration to allow for comparisons between studies [57]. These concerns have led to the development of “micro” shear and tensile tests wherein specimens are significantly smaller [57]. Though not entirely free of the problems mentioned above, the microtensile testing of the bond between the adhesive and the tooth is also simpler to conduct than other tests of interfacial properties, both in terms of specimen preparation and the tools to conduct them, and thus both “macro” and “micro” tests remain popular [57,59].

**Figure 6:**
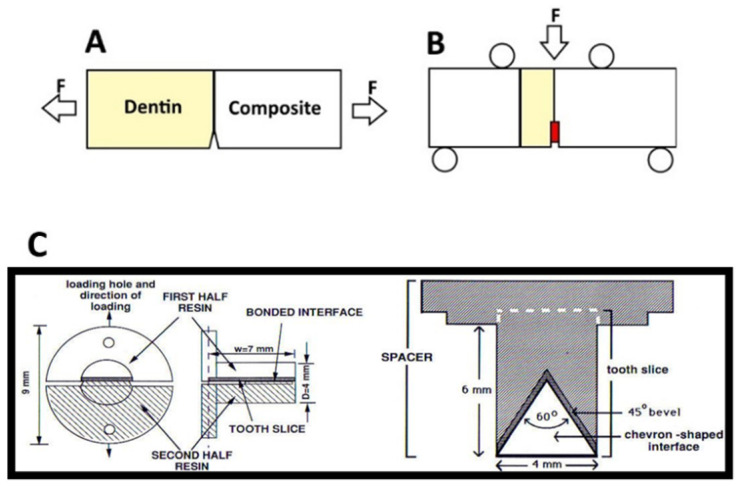
Simplified Specimen geometries for (**A**) a single edge notch beam test conducted. F represents the direction of force exert on the specimen. Modified from [54]. (**B**) An example of a single edge notch chevron beam test; the chevron created at the resin dentin interface is colored in red. Modified from [55]. (**C**) short rod chevron test, modified from [38].

Due to the possible variability in bond strength, due to differences in specimen geometry and unequal stress propagation, it may be best to test the material’s resistance to crack propagation or initiation; effectively, its resistance to fracture or fatigue [10,57]. The formation of a bonded interface can be considered to be imperfect, with flaws inherent in the process of curing [60], and it is the formulation’s tolerance of these flaws that is most likely the key to restoration longevity. These measured values should be more independent of geometry or testing modality. It is for this reason that the analysis of the fracture mechanics of bonded interfaces is seen as a valid means of measuring changes in the adhesive [59,60]. The plane-strain fracture toughness (K_IC_) is one such variable, measuring the critical stress value at which a crack will propagate and, by extension, the interface will fracture [55]. The testing of bonded interfaces is highly complex and variable, with many different types of specimens, and methods of testing; however, the most common methods of testing are the variations of the single edge or chevron-notched tests, and short rod Chevron notch tests in order to calculate K_IC_ for resin composites (Figure 6) [61]. Such stress to fracture tests, wherein the load is slowly increased until the material or interface fractures, are common. However, these methods have been criticized primarily due to the observation that this kind of steady and unending increase is not representative of the clinical situation [62]. This is somewhat of a mischaracterization of the intent of these tests; the results are still valuable, as they are used in directly determining changes in the breakage resistance due to the biochemical degradation that causes interfacial flaws, as they can be used to represent the integrity of the bonded interface after aging [63].

In the context of the calculation of the fracture toughness of the bonded interface, variations of the single edge notched beam test (Figure 6A,B) and short rod chevron tests have been commonly employed (Figure 6C) [61,64]; however, an interface presents unique challenges in a fracture mechanics approach due to the complexity of stress propagation [59]. Of the two popular tests mentioned above, beam-based tests may be more difficult; though the geometry of the specimen itself is relatively simple [61], the specimen must be larger to meet linear elastic fracture mechanics requirements. The length of the loading span should be about 10 times greater than the thickness of the specimen [55]; this can be difficult to achieve when limited by the size of teeth, and/or expensive due to the use of large amounts of resin composite required to complete the specimen and even out the ends, as seen, for example, in Munck et al. (Figure 6B) [65]. In addition, the notch or chevron must often be sawed into the specimen, and this may introduce added error or variability [55,59,61]. In contrast, the short rod chevron test can be made entirely with molds, with no post cure interference required [42]. Across many different kinds of bonding agent, the unaged bonded interfaces of the composite and dentin generally have fracture toughness values ranging from 0.25 ± 0.12 to 1.11 ± 0.2 MPam^1/2^ [59], or more.

Instead of directly determining the mechanical properties of a given material, one can also attempt to measure the fatigue resistance of the bonded interface. This is related to the K_IC_, as a material with high fracture toughness is more resistant to subcritical crack growth caused by fatigue [66]. These alternative methods of testing that more closely mimic in vivo mechanisms of failure instead utilize cyclic loads until the specimen fails. Therefore, cyclic loading tests may be seen as more representative of clinical stresses and failure as they mimic the repeated tooth contact and associated mechanical stresses of the in vivo environment [36]. A clinically normal bite force is often considered to be approximately 70 N, but that being said, the load per tooth in the mouth depends on a multitude of factors including the number of teeth, habitual bruxism, and other factors [33]; there is also no consensus on the parameters of a protocol [66]. There are many different possible techniques that involve such cycling, and many different possible specimen geometries (again making reporting the specifics of test and specimen protocols important) but the fatigue resistance of a given material is often determined through a probabilistic approach, documenting the number of cycles before each interface’s failure, and examining the chance of an interface surviving as a function of a cycle number at a given stress amplitude, or determining the value of stress that a sample can survive for a given number of cycles [32,66]. The key parameters are, therefore, the level of stress, the cycle number, and the rate of cycling. It has been found that 10.5 MPa is the stress consistent with the occlusal forces on a molar, while 0.5–3 cycles per second and approximately 10^5^–3 × 10^5^ cycles in a year are the generally accepted parameters for cycling [33,34,67]. The specimens used in this testing are also generally beam specimens, placed in four point flexure similar to those seen in Figure 6B [32].

Another valuable avenue of analyzing the degradation of the interface is the use of fractographic analysis of broken specimens to determine the point of failure. Specimens, once broken, can be examined under a microscope to determine where the critical crack began, whether in the composite, dentin or the adhesive [68]. This allows one to discern changes in crack initiation in total- and self-etch interfaces as the material ages [42,54].

## 5. In Vitro Simulation of the Oral Environment

### 5.1. Mechanical Simulation

The key parameters in a mechanical aging protocol that simulates the everyday use of the mouth are as follows:

(1) the maximum load the sample must withstand;
(2) the number of cycles simulating the forces within the mouth;
(3) how long each cycle lasts;
(4) the manner of contact with the specimen; the horizontal and/or vertical path of the load.

Each of these parameters can vary significantly from study to study; for example, with maximal loads between 30 and 250 N; many different cycle numbers, from 50,000, up to 5 million (rough estimates for a month to a year of testing); a large variety of cycle frequencies (though 0.5–3 cycles per second is generally considered clinically representative [45,67]); and the direction of force application or load is often not mentioned [62]. Collectively, this makes comparison between studies difficult if not impossible.

### 5.2. Chemical Aging Simulation

One of the simplest ways of chemically aging a specimen is by storage in water or another medium. Different media can be used for this purpose, with some commonly used substances being water, ethanol, and aqueous solutions of sodium hypochlorite [69,70,71]. All of these have shown efficacy in degrading bonded interfaces; however, they do not simulate the enzymatic activity of saliva [45]; in addition, constant incubation in ethanol or sodium hypochlorite are not circumstances that occur in the oral environment, making drawing analogies in the aging of the material in vivo from such storage media difficult.

In an attempt to more closely simulate the enzymatic activity of saliva, processed human saliva has also often been employed [42]. However, the enzymatic activity of saliva is short lived, necessitating frequent replenishment, and the collection of human saliva is difficult to conduct; therefore, artificial saliva can be used instead. The activity of salivary esterases is analogous to the activity of esterases such as cholesterol esterase (CE) and pseudo cholinesterase (PCE) [72,73]. With this analogous activity in mind, an artificial solution can be created that replicates the in vivo biochemical resin degrading activity of saliva against a particular substrate. Incubation in this simulated human salivary esterase (SHSE) has been shown to increase the rate of bisHPPP release from bonding agent materials in comparison to incubation in buffer and can match the degradation produced by saliva [18,54] and has been found to decrease fracture toughness [54], linking the chemical degradation of the bond to its mechanical degradation. Shokati et al. found a significant decrease in fracture toughness after 180 days of incubation in human salivary derived esterase, from approximately 0.86 ± 0.16 to 0.55 ± 0.13 MPam^1/2^ [42]. In addition, incubation in salivary esterase or simulated salivary esterase has been found to increase the release of BisHPPP, implying that the esterases catalyze the reaction [18,42].

### 5.3. Caries Formation and Propagation

As secondary caries is such a prominent cause of the failure of resin composites, it stands to reason that the materials, bonded to prepared tooth samples to make a specimen, must be aged in cariogenic conditions to determine the resistance of the material to the acidic, degradative conditions when dental caries is formed. 

The simplest method of studying this process is to simply simulate the conditions that prompt the formation of dental caries, by placing a restored tooth specimen into an acidified solution and monitoring the demineralization of the enamel or dentin and studying the specimen post-demineralization to determine if the bond has deteriorated. Since cariogenic bacteria form lactic acid, buffered lactic acid has been the most common media used as it has been found that this system produces lesions similar to those formed in vivo [74]. A more complex form of this protocol would be to cycle the pH, thereby simulating the in vivo cycles of demineralization and remineralization dental caries undergo in the mouth by transferring the samples between acidic and neutral, remineralizing solutions [75].

Rather than simply simulating the conditions that lead to the formation of dental caries, one can also directly create dental caries similarly to oral conditions, through the incubation of a specimen in media that contains a bacterium such as *S. mutans*. Since caries is typically caused in a small region of the tooth where the microenvironment becomes acidic due to the release of acidic by products of bacterial carbohydrate metabolism, this more directly simulates the process. One drawback is that pH cycling is not typically possible, as surface remineralization under the biofilm cannot occur as it does in vivo due to fluctuations in the biofilm’s metabolic activity [44]. Though mono-species models are simpler to conduct, *S. mutans* is only one species in a highly complex microbial community, limiting such models [76], and making multispecies biofilms somewhat more representative of the oral environment, though ensuring the inclusion of appropriate species and determining the proportions of included bacteria can be a difficult process [44]. Multispecies biofilms have been found to be both larger, and more capable of causing enamel demineralization [77]; though primogenitor bacteria such as *S. mutans* are early colonizers that lay the foundation for dental caries, they are not the be all and end all in this process, wherein caries is the result of the interactions between many species. In a study by Shu et al., multispecies biofilms achieved a minimum sucrose-induced pH of 3.66 ± 0.10, while monospecies biofilms achieved a minimum pH of 3.90 ± 0.08 at lowest, and had a higher capacity to soften enamel in comparison to monospecies biofilms [77]. Biofilms that incorporate carefully chosen species of bacteria allow for a great deal of control and standardization between experiments, though lesions do not form as they do in vivo, and the pH cycling that occurs in the oral environment cannot be conducted in these tests as mentioned above.

Building upon multispecies biofilm models, the method that most closely simulates the oral environment is to directly sample the oral microbiological environment of a human volunteer—this is referred to as the microcosm model. This model is rather unwieldy though, because the great diversity of species involved makes it difficult to parse out how individual species are involved in the process [44], and of course the bacterial community will vary significantly from person to person, making control difficult.

**Figure 7:**
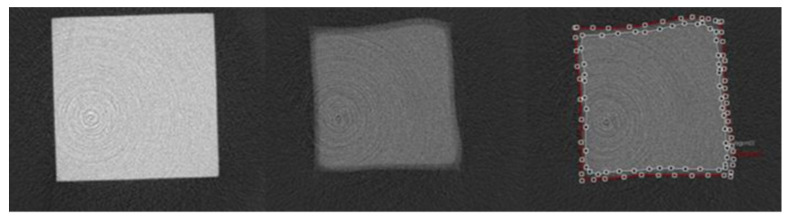
Axial view of micro computed tomography images within the resin–dentin interface of representative total-etch resin–dentin bonded specimens after 7 days of incubation in media containing *S. mutans* and *L. rhamnosus*. **Left**: µCT image of intact resin, 21.25 µm above the interface; **Middle**: µCT image of demineralized/cavitated dentin, 42.5 µm below the interface; **Right**: µCT image of interface with areas of demineralization (white line) and cavitation (red line) [78].

The cavitation left by the progression of these caries can then be quantified by several methods, and the architecture of the biofilm can be characterized by confocal laser scanning microscopy as noted Kermanshahi et al. [26]. A simple method of measuring the progression of the lesion is by measuring its depth [74], while the demineralization of enamel and dentin can also be measured through the use of microhardness and microradiography to determine the damage caused by the overlying biofilm [77]. Another method is the use of microCT to quantify cavitation and demineralization in a beam specimen after aging in a bacterial or acidic media (Figure 7) [78].

## 6. Accelerated Aging

Chemical degradation by storage may not successfully fulfill the ideal quality of in vitro tests; that of faster results than those that could be obtained from a clinical trial. One problem is that many tests take significant time to conduct adequately. The time spent incubating samples in distilled water or simulated saliva solutions can vary from months [46,54] to up to 6 years [69]. This partially neutralizes one of the primary reasons for in vitro testing; that of speedy results.

Thus, accelerated aging is often a necessary consideration in in vitro testing. The cyclic loading used to simulate the mechanical stress of the oral environment, as discussed above, could be considered an accelerated aging protocol as it is meant to compress the normal mechanical stresses of the tooth into a shorter time period. As mentioned above, for example, the compression of a year’s worth of occlusal loading (10^5^ cycles) into days or weeks with the help of a machine will greatly accelerate the study [79].

Thermocycling is another common form of accelerated aging, which works by compressing the in vivo temperature cycling of the oral environment (during eating, breathing, etc.) over the course of years into a shorter time. Some work has been conducted to determine the parameters that an accelerated aging protocol should follow when using a thermocycler—for example, it has been estimated that the maximum intraoral temperature generally achieved is 45–47 degrees Celsius (when drinking a hot liquid that does not cause pain), while the minimum is approximately 5–10 °C (cold food and drink) [80,81,82]. Though other extremes, from 0–67 °C [83], have been recorded and proposed, they may not be entirely representative of everyday temperature variation. 

A common problem faced by both methods is standardization between studies, or rather, lack thereof. Standardization would allow for comparison between studies. With regard to thermocycling, the mouth is estimated to cycle between highs and lows roughly 50–80 times a day, or about 10,000 times over the course of a year [80]. However, these numbers remain estimates, and there is disagreement between studies on the dwell time, the number of cycles, and very little work has been performed on the equivalences between protocols and real time incubation/degradation [12]. To underline this controversy, the ISO standard for testing dental materials is 500 cycles between 5 and 55 degrees Celsius in order to examine the durability of an adhesive bond [84]; in comparison to the suggested figures, 500 cycles seems far too small a number to notice a significant change in the material, whether mechanical or chemical. Cyclic loading has similar controversies regarding protocol, with little consensus, as discussed above [62,66].

## 7. Synthesis of In Vitro Aging Protocols

Since the oral environment is a confluence of multiple factors, wherein a complex interaction between bacteria, inherent flaws, and chemical and mechanical degradation all act together to compromise the hybrid layer, some in vitro aging protocols have attempted to combine multiple different stresses to create an “artificial mouth”; these have found a high degree of correlation with clinical forms of failure, but these have focused primarily on mechanical degradation, rather than other sources [85,86]. Combining accelerated aging from mechanical loading or thermal cycling with chemical degradation, by, for example, thermocycling specimens while they are immersed in simulated human salivary esterases has not been thoroughly examined and could stand to be an intriguing avenue of research.

In fact, thermocycling and mechanical cycling protocols are usually carried out in a storage medium, such as water, artificial saliva or other substances [36,87]. However, many of these protocols can be vague with regard to the exact composition of an artificial saliva solution if it is used; it is difficult to know if these are only replicating the molecular concentrations of salivary compounds or using enzymatic salivary solutions. Given the evidence that salivary enzymes greatly accelerate the chemical degradation of methacrylate resins [54], it stands to reason that accelerated protocols using such formulae should be developed, as saliva may serve to exacerbate other forms of degradation [26].

Another difficulty is that some aspects of in vitro testing may be impossible to conduct simultaneously with others—for example, the incubation of a sample with bacteria makes pH and temperature cycling over many days untenable [44]. In addition, the interactions of bacteria with salivary or simulated salivary esterases is unpredictable [26,27].

To compensate for this difficulty of incorporating bacteria into a hypothetical artificial oral environment, protocols may be able to compartmentalize multiple methods of degradation into different phases of aging, for example, Kermanshahi et al. and Huang et al. examined the penetration of bacterial biofilm into the marginal gap after incubation in SHSE [26,27]; specimens were first degraded in the simulated saliva, and then exposed to bacteria in the required environment. Though this does not combine all of the factors into a single phase, it may be a suitable compromise in order to expose the interface to a multitude of different forms of degradation.

## 8. Conclusions

Despite their popularity, dental methacrylate resin restorations are vulnerable to fatigue and degradation that shortens their service life significantly. The hybrid layer is the weak link of the restoration, and thus, new bonding agents are of especial importance; however, in order to develop new materials and methods to make the bonding agents more resistant to chemical and mechanical degradation requires in vitro tests that can more quickly determine the material’s properties, aging, and resistance to degradation. These in vitro tests, however, often take significant amounts of time, or are not standardized, making comparisons between them difficult. Work remains to be performed to correlate accelerated tests with real time aging, and to create tests that combine the various mechanisms of hybrid layer degradation in order to create a more complete simulation of oral conditions. Though such protocols could be difficult to create due to the difficulties in integrating the different aspects of degradation, combining chemical degradation that replicates salivary incubation with mechanical or thermomechanical challenges is an intriguing avenue of study, serving both to combine different kinds of degradative stress, and potentially working to accelerate study, making in vitro testing both quicker, and potentially more representative.

## Data Availability

Not applicable.
